# The Organic Diseases of the Urethra

**Published:** 1842-04

**Authors:** 


### ART. II.

1. *De Organische Gebreken der Urethra, beschreven door* D. J. A. ARNTZENIUS, Med. et Chir. Doct.—*Utrecht,* 1840. 8vo, pp. 442. Met Platen xvi. Folio.

*The Organic Diseases of the Urethra, described by* Dr. J. A. ARNTZENIUS, Doctor of Medicine and Surgery.—*Utrecht,* 1840. With 16 Plates in folio.

2. *A Treatise on Stricture of the Urethra, containing an account of Improved Methods of Treatment; with an Appendix on Dilatation by Fluid Pressure in the Treatment of Urinary Calculus and other Diseases.* By JAMES ARNOTT, M.D., late Superintendent Surgeon in the Honorable East India Company's service. Second Edition.—*London,* 1840. 8vo, pp. 234.

3. *Practical Observations on the Pathology and Treatment of Stricture of the Urethra, with Cases.* By ROBERT WADE, Senior Surgeon to the Westminster General Dispensary.—*London,* 1841. 8vo, pp. 149.

4. *Observations sur les Rétrécissements de l'Urètre par cause traumatique.* Par J. FRANC, Professeur agrégé à la faculté de Médecine de Montpelier, &c.—*Paris,* 1840. 8vo, pp. 210.

*Observations on Contractions of the Urethra, consequent on wounds, and on their treatment.* By J. FRANC, Professor in the Medical School of Montpelier.—*Paris,* 1840.

5. *Practical Hints on the Treatment of Stricture of the Urethra.* By BRANSBY COOPER, F.R.S. (*Guy's Hospital Reports,* Vol. V. 1840.)

IT is our intention, in the present article, to give some account of the present state of our knowledge respecting strictures of the urethra generally; and in doing so we shall arrange, in our own fashion, the materials supplied by the works before us under the different heads into which the subject naturally divides itself. At the end of the article we shall state our opinion of the character and merits of the respective works.

*Anatomy and relations of the Urethra.* Dr. Arnott prefaces his treatise with a general view of the anatomy and relations of the urethra. "The urethra arises from that part of the bladder which in the erect position of the body is the lowest, and may be considered a tubular prolongation of its inner coat." (p. 2.) This is true only to a certain extent, and at a certain period of life. The bladder is a moveable viscus, and its relation to the arch of the pubes, (to which its membranous portion only is fixed by the triangular ligament,) is constantly varying from the condition of the surrounding viscera, and from the influence of age. In the fœtus the bladder is placed almost entirely in the abdomen, and above the pubes, the pelvis not being, as yet, sufficiently developed to admit of its descent. The rectum at this period, distended with meconium, and filling the whole of the imperfectly formed pelvis is another obstacle to the descent of the bladder to the position it subsequently occupies. As the bones of the pelvis acquire with age an increase of size, and become more and more developed, so the bladder is found lower and lower in the pelvis, till, in old age, it is even in a moderately distended state almost totally concealed behind the bones of the pubis. These anatomical facts show that Dr. Arnott's description of the opening of the urethra into the bladder can only relate to the earlier periods of life; in adult and old age, there is always a pouch of greater or less size formed behind the urethral opening into the bladder.

So well aware was the late M. Dupuytren of this situation of the bladder in infancy, and at the earlier periods of life, and also of the occupation of the pelvis by the rectum at this age, that he used to give the following directions as necessary to be observed in the performance of the lateral operation for lithotomy in young children: It results from this disposition, that if a knife is attempted to be plunged into the bladder, or drawn from it in a horizontal direction, the instrument will, in the first instance, pass between the bladder and the rectum, and penetrate the latter; and in the second, after having divided the neck of the bladder, and prostate, at this period very small, the instrument wounds the intestine longitudinally. From which it is easy to conclude that, in order to avoid the rectum in children on whom the operation for lithotomy is performed in the ordinary situation, it will be necessary to give to the knife a direction parallel to that of a line which, setting out from the umbilicus, shall terminate at the tuberosity of the ischium of the left side; and consequently, that the handle of the instrument must always be more depressed, and the point more raised than in the adult.

*The causes of stricture of the urethra.* These are of varied kinds, but inflammation affecting its mucous and submucous cellular coats is the chief agent in all. Mr. Wade enumerates as causes: 1st, protracted gonorrhœa; 2d, continued chronic urethral inflammation in persons possessing an irritable urethra; 3d, the continued discharge of unhealthy urine containing the lithates or phosphates in excess; 4th, calculi or other causes producing irritation and inflammation of the kidneys, ureters, or bladder, subsequently extending to the urethra; 5th, external or internal injuries, and tumours. To this last class of causes M. Franc's work is exclusively devoted; and we may add to those which Mr. Wade has enumerated the cicatrices consequent upon the healing of chancres situated in the urethra. We are not aware that this has hitherto been noticed, but we have in our own practice had occasion to witness two or three instances where a diminution in the caliber, and a total destruction of the elasticity of the urethra have originated from the healing of chancres situated in the urethra itself. Dr. Arnott believes that strictures are generally caused by *irritation* of the urethra, " produced either directly or by sympathy with some contiguous parts under disease." (p. 33.) He brings forward as examples the passage of pieces of gravel along the urethra, the unskilful passing of instruments, the presence of a stone in the bladder, affections of the rectum, &c.; but he thinks that gonorrhœa is a cause so much more common than any of the others, that it may be almost considered the sole one. Hunter, it is well known, believed that strictures were never produced by gonorrhœa. Many modern surgeons, as Abernethy, Sir E. Home, Desault, and others, believed and taught that strictures were generally produced by the employment of injections during the inflammatory stage of gonorrhœa. We have no hesitation in confidently declaring that we are much more likely to expose our patient to the chance of stricture by totally omitting the use of injections in gonorrhœa than by their judicious employ. The quicker a gonorrhœa is cured the less likely are we to have stricture follow it. Gonorrhœa is a disease whose ill consequences are in a direct ratio with the period of its continuance. Cure it quickly if you can, but at the same time cure it completely. If a spot of chronic inflammation remain, however trivial it may appear in the commencement, it will slowly thicken, and destroy the elasticity of the urethra. These are the diseases which, in the language of Desruelles, "youth entails upon old age."

"In general, strictures of the urethra arise from an inflammation, the consequence of some irritation often syphilitic (i. e. gonorrhœal), of which the mucous membrane is the seat, and which is not resolved, but assumes a chronic form, and perhaps there is effused therein a plastic material which then not only gives rise to the various forms of strictures depending on the mucous membrane itself, but being at the same time effused in the subjacent tissue produces also the unnatural hardness. In cases in which this inflammation is limited to one point of the mucous membrane, this swells up, its connexion with the subjacent cellular tissue is loosened, it becomes wrinkled, and a fold is formed on its surface which is continually enlarged by the pressure of the urine, and constitutes the so-called *bridle;* the mucous membrane contracts throughout a certain extent of the length of the urethra, and forms the long stricture. The mucous membrane itself commonly remains in these cases smooth, and apparently healthy on its inner surface, and Sir C. Bell, amongst others, has, in our opinion, committed an error in comparing the formation of these strictures with that of false membranes on the pleura in cases of pleuritis. On mucous membranes this exudative inflammation and the formation of organized false membranes are by no means so common as on serous membranes. On the surface of the mucous membranes on other parts of the body, we see altogether different results produced by chronic and especially by catarrhal inflammation; they become villous and undergo various degenerations, which are chiefly the consequences of ulceration to which the mucous membranes are particularly prone, but growths are not commonly produced by means of newly-formed tissues. We believe also that the polypous vegetations or fleshy growths mentioned by many writers, must be ascribed to ulceration of the mucous membrane of the urethra, which may then give rise to a variety of growths and degenerations, and sometimes to perforation of the urethra. On the contrary, we should regard it as certain that the indurations which are so often observed in the neighbourhood of strictures, are by no means proper to the mucous membrane. In these cases the inflammation is imparted to the subjacent tissue, whose cells become filled with plastic substance which is organized around the mucous membranes of the urethra, and whose tissue thus loses its elasticity and often acquires a fibrous or even cartilaginous hardness. Thus there is produced at once a diminution of the diameter of the canal, and a decrease of its flexibility and aptness for extension, which hinder the discharge of the urine, erection, and ejaculation of the semen. In bridles therefore or band-shaped strictures, in long strictures by contraction of the mucous membrane, and in polypous or fleshy excrescences, the mucous membrane itself is the seat of the disease; in callous or cartilaginous induration, the change of structure lies rather in the subjacent tissues. How far ulceration of the mucous membrane is the cause or the effect of the stricture is a question not satisfactorily solved. We have almost always seen it behind the stricture, and have therefore regarded it as the consequence. And probably ulceration is often assumed when it does not exist, for the discharge of purulent matter, so common in strictures, is far from proving its presence. . . . . That abscesses and even ulcerations often occur in the neighbourhood of, and especially behind, strictures, and that the latter at last perforate the wall of the canal, and give rise to various irremediable consequences, does not admit of doubt; but it is another question whether these are not the result of the obstructed passage of the urine and of the inflammation which is kept up behind the already-formed stricture, and which gives rise to the muco-purulent secretion and the ulceration." (Arntzenius, pp. 146-50.)

The principal causes of traumatic stricture are, 1st, contusions with or without solutions of continuity of the urethra in the perineal or scrotal regions; 2dly, lesions, with or without solutions of continuity, of that part of the canal which forms part of the penis; 3dly, internal lacerations resulting from the careless introduction of catheters, &c. or effected by calculi, or their fragments passing from the bladder. The majority of traumatic strictures of the urethra are situated anterior to the subpubic ligament, but they are sometimes met with behind this. That part of the urethra most commonly affected is at the anterior part of the perineum, and the interval between the two sides of the scrotum.

The importance of strictures resulting from injuries of the urethra depends much upon the degree of injury which has given rise to them. These injuries may be contusions, lacerations, or loss of substance. In connexion with them also are to be considered the injuries to the neighbouring parts. *Contusion* may be followed by general swelling and temporary stricture, ceasing when the swelling disappears. Induration and consequent stricture may follow the inflammation which is produced by contusion simply. *Laceration* may occur without loss of substance. It is always accompanied by swelling, and is generally followed by fistulous openings. It is not unfrequently succeeded by induration and consequent stricture. *Loss of substance* may occur in any situation and in all degrees, the character of the stricture being much influenced thereby.

Mr. Wade classes strictures under the following heads: 1, The dilatable stricture; 2, the simple chronic stricture; 3, the impassable stricture; 4, the irritable; 5, the inflammatory; 6, the stricture with marked disposition to contraction; 7, the spasmodic; and 8, the stricture from external or internal injury. The nature of several of these varieties, however, appears to be the same, their difference being merely in degree. Thus most permanent strictures exhibit a marked disposition to contraction: we think the division of strictures into the permanent, the spasmodic, and the irritable, sufficient for all practical purposes; it is good inasmuch as it indicates a real difference in the nature of the stricture, and one which requires for each variety a distinct peculiarity in treatment. The following observation by this author is worthy of notice:

"Irritable strictures are usually observed either in persons whose digestive organs are in an unhealthy state, or in those whose general health has been impaired by residence in warm climates; or who have been accustomed to indulge in the pleasures of the table, by which a high degree of excitability of the nervous system has been produced. In the lower classes of society, the irritability is most commonly brought on by the use of ardent spirits. An irritable stricture, if under the influence of muscular action, is much disposed to spasm; and if in a situation where there are no muscles, the irritability caused by the pressure of the instrument is often sufficient to occasion such a distension of the vessels of the corpus spongiosum around the obstruction, and of the lining membrane at the seat of the disease, as frequently, for a short time, to obstruct entirely the passage of the urine." (Wade, p. 20.)

Amongst the authors before us there appears to exist a difference of opinion as to the mode in which the term spasmodic stricture should be applied. Dr. Arnott says, "spasmodic stricture depends entirely on excessive or spasmodic contraction of some of the urethral fibres, and exists no longer than that contraction continues." Of course this explanation will not be admitted by those who deny the existence of muscular fibres around the urethra anterior to the bulb. We are more disposed to sanction the explanation given by Mr. Wade. The term spasmodic stricture, he says, "should be confined to an obstruction at the bulbous or membranous portions of the urethra from involuntary contractions of a muscle or muscles surrounding that canal."

"Spasmodic stricture must certainly be considered as very generally the result of inflammation at the membranous or bulbous portions of the urethra, causing so much irritation, especially when the urine passes over the inflamed surface, as to produce contraction of the surrounding muscles, so that their necessary relaxation on contraction of the detrusor is prevented. These inflammatory spasmodic strictures most frequently occur in persons who, when affected with gonorrhœa, indulge in some excess or other, especially in wine, spirit, or punch; the last the most injurious of all, from its admixture with acid: or they may arise from the sudden suppression of the gonorrhœal discharge from cold or other causes. In these cases, the gonorrhœal inflammation, rapidly extending to the bulbous and membranous portions of the urethra, often excites so much spasmodic action of the surrounding muscles as to cause complete retention of urine; or the same effect may arise from the sudden increase of preexisting inflammation in those parts. All permanent strictures at the bulbous or membranous portions of the urethra may be increased by spasmodic action brought on by the passage of instruments, injurious indulgence in vinous or spirituous liquors, or indeed any cause which may excite irritation and inflammation at the seat of obstruction." (Wade, p. 24.)

*Treatment.* The works before us advocate several modes of treatment; by simple dilatation, dilatation by fluid pressure, and by the bougie armed with potassa fusa or the nitrate of silver. Each of these methods of treatment is applicable in various cases, and we shall lay before the reader those circumstances in which each may find its advantageous employ.

The treatment by simple dilatation by means of bougies, is the oldest and most simple method of treating strictures of the urethra.

In speaking of the history of bougies, Dr. Arntzenius introduces an interesting fact lately published by Dr. N. C. Bussemaker, in Dr. Heije's "Wenken en Meeningen:"

"The introduction of instruments into the urethra in the treatment of its strictures," he says, "dates from the earliest periods of the Christian era, and an important passage has lately been made known, which was written by Heliodorus, who lived in the beginning of the second century, and which is to be found in the fiftieth book of the Collectanea Medicinalia of Oribasius, published by Angelo Maio. It leaves not the least doubt that the plan of dilatation, by means of bougies made of papyrus, lead, tin, or quills, was, even at that early period, in use." (Arntzenius, p. 68.) *

* The following is a translation of portions of the passage from Heliodorus, quoted by Dr. Cato Bussemaker. "The passage for the urine, in consequence of ulceration, closes up with flesh. It does not altogether close, but only at some part of it, where it either becomes narrow or is completely filled up by flesh. When this takes place the patient makes water with difficulty or only by drops." Then, he says, there may be complete retention of urine, and he describes a rough mode of cutting into the bladder and destroying the carnosities for its cure, and says that in order to keep open the urethra it should, "especially during the first days after the operation, be dilated by introducing a compressing instrument made of dried papyrus." For making these instruments, "the papyrus must be macerated for two or three days, then placed on a cylindrical tube and fixed tightly round it . . . . . it must then be left to dry as completely as possible, and

There can be little doubt that this is the earliest record of the employment of these instruments. Hippocrates indeed mentions the use of tin tubes in the treatment of fistulæ, but nowhere speaks of applying them to the diseases of the urethra; and it may be reasonably believed that the instruments, more or less resembling catheters which have been dug from the ruins of Pompeii, were also employed in the former class of affections.

In the same section, after treating at considerable length of the several kinds of bougies invented for different intentions, Dr. Arntzenius says that, for the purpose of exploring the urethra to determine the degree, the kind, and the extent of a stricture in it, he has found no instruments so good as some manufactured by Van Gesscher of Amsterdam.

"They consist of a strip of parchment rolled up so tightly that its layers adhere pretty firmly to one another, and which has at one end of it a knob or drop of glue made of boiled parchment. This knob contributes very much to the facility of introducing the instrument into a stricture; one can feel it slipping in; and then, by the heat of the urethra, it melts and the bougie unrolls of itself, while the melted glue is retained without injury to the urethra and does not possess the slightest degree of any irritating quality. Such instruments may be manufactured of very small size and yet possess sufficient strength; and their unrolling is rather advantageous than injurious in consequence of the slow dilatation to which the stricture is thus subjected." (p. 99.)

Their cheapness is mentioned by the author as an additional recommendation of these bougies, and this is not an unnecessary quality, since in general each can be used only once. Whether they deserve all the praise bestowed on them experience only can decide; to those who are disposed to try them we can give our evidence that the author does not seem one accustomed to speak rashly or to recommend unjustly, and that in general the bougies with knobbed extremities, so highly commended by Sir Charles Bell, are, of all, the most useful for the purpose of exploring the urethra, whateverM. Ricord may say to the contrary.

There are three modes in which the treatment of dilatation may be performed: the first, which is most commonly practised in this country, is that of gradual dilatation by bougies of various kinds, the size being slowly increased. The second is the forced dilatation of Mayer and Lallemand, to which we need not more particularly allude, its dangers being such that it is not likely to be practised by any prudent surgeon in this country. The third is the fluid dilatation of Arnott. Dr. Arntzenius gives the preference to the treatment by simple dilatation. He supposes two different conditions in which patients may present themselves for treatment; in the first, a bougie can be passed into the stricture; in the second the canal is apparently completely closed and there are urgent symptoms of retention of urine. (p. 358.)

then, while still fixed on the tube, having been bent into the form of the urethra, may be introduced into it. Around the penis there is to be placed a sponge wet with cold water," and with proper bandages, the patient is to be thus left till the third day. Then "water is to be injected into the urethra to cleanse the ulcer, and if necessary another *pressorium* instrument is to be introduced, so as to dilate the canal still more. . . . An anti-inflammatory plaster is now placed round the penis, and it is bandaged and raised up in the usual manner. But after the fourth day, instead of the tube, a tin or leaden instrument is introduced carrying a little shield (*parmulam*), so that cicatrization may be brought on at the same time that the passage is dilated." (Heije's Wenken en Meeningen. Deel i. bl. 37.)

In the former case, the first step is to introduce one of the parchment bougies already described into the bladder; or, in the few cases in which the stricture is so long that these are too soft or become too soft in their passage to go through it, then to introduce one made of firmer materials or a catheter. The smallest ivory bougies are rarely strong enough for this first purpose, but when the canal has been somewhat dilated they are particularly useful, and especially from the very slight irritation which they produce in even the most sensitive canals. An instrument having been once passed, one should subsequently be introduced daily for several days, and allowed to remain in for a quarter of an hour or more up to an hour, according to the pain it produces. For the first few days the size of the instrument must be very cautiously increased; but afterwards the increase may be made with less danger, and the periods of introduction be made gradually more distant, till the operation is not performed more than once in a month, and then with a full-sized instrument of either wax or softened ivory. The only objection to this mode of treatment is its slowness; but this the author holds is more than compensated by its safety and the security against a return of the disease, which it affords in a far greater measure than any rapid mode of temporary cure.

But there are cases in which this method cannot be followed, 1st. When the urethra is very irritable and sensitive. In these, antiphlogistics should precede the use of instruments, and then, at first, only the softest should be employed, and these should only be introduced down to the stricture and held there for some time before an attempt is made to pass one through. 2d. In long and indurated strictures the use of bougies will produce only a certain amount of benefit, and then neither dilatation nor mechanical irritation will clear away the narrowed part. In these the most useful means are bougies with their ends smeared with nitrate of silver or corrosive sublimate. 3d. In hard bridles close by the external orifice of the urethra, which obstinately resist the passage of a large bougie; and in these, almost exclusively, the author considers incision admissible. The operation may be performed with an ordinary probe-pointed bistoury, unless the bridle lie more than two inches from the orifice, and then some one of the numerous stiletted catheters must be employed.

In proposing the employment of dilatation by fluid pressure, Dr. Arnott passes in review the inconveniences and evils likely to arise from the use of the simple bougie. In noticing the rationale of the mode of cure effected by these instruments he contends, and we think with great reason, that the cure is seldom permanent. The first effect of the introduction of a bougie sufficiently large to pass through a permanent stricture is violent distension of the diseased part of the urethra, which, if the instrument can be left in the canal, gradually diminishes by dilatation or absorption taking place; so that at last the instrument remains in the canal without any distending action. "After this its longer continuance in the passage can be of little avail, but may prove hurtful by the irritation excited. In curing strictures by the bougie, it often happens, from the increased tendency to contract, that, in the intervals between the introduction, whatever is gained by one is nearly lost before the succeeding, and this interruption to the process of distension, with the irritation produced by the so frequent passing of an instrument, is sufficient to account for the ordinary tediousness of the cure." To obviate, in some measure, the evils attendant on the use of bougies of a fixed size, surgeons have had recourse to the employment of instruments composed of substances which swell by heat and moisture, and thus become enlarged when suffered to remain for a certain time in the urethra; tents made of sponge, and bougies manufactured of catgut, leather, &c. &c., have been devised for this purpose. Dr. Arnott's fluid dilator was constructed with the same views, viz., the capability of being enlarged when passed into the strictured part of the urethra.

Dr. Arnott states that a dilator for the cure of strictures should be of little bulk, and of easy introduction, and should be made capable of assuming and retaining any shape and magnitude when in the urethra; it should also be capable of exerting a distending force to any degree, and always under control. Such are the properties and advantages assumed by Dr. Arnott for his fluid dilator. It consists of "a tube of oiled silk, lined with the thin gut of some small animal to make it airtight, and attached to the extremity of a small canula, by which it is distended with air or water from a bag or syringe at the outer end, with a stop-cock or valve to keep the air or water in when received."

For the particular description of the whole apparatus, we refer the reader to Dr. Arnott's treatise.

"The only part of the preparation of the dilator requiring nicety of execution, is the attachment of the distensible tube to the conducting tube and wire, which must be at once very neat and very secure. The silk tube and lining gut should be tied on together, the artist taking care that the wire be kept exactly in the axis of the tube, or that the wrinkles or folds at the extremity be equal all round. The tyings may be made conical by notching the extremity of the silk, after two or three turns of the small strong waxed silk thread have been made round it, and by then continuing the thread completely over it. The tyings should then be smoothed by a coating of bougie wax, and if unvarnished silk has been used, the operation is completed by covering both the silk and the tyings with a bit of gut. The secure attachment of the distensible tube to the canula and wire is a matter of great importance; for, should the silk become detached in the canal beyond the stricture, it might happen that the combined action of the urethra and flow of urine would not be able to expel it, until the stricture were fully dilated; it behoves therefore the instrument maker and the surgeon, to be careful that there exist no such hazard." (pp. 227-8.)

The distance from the orifice of the urethra of the stricture to be dilated must be first measured by a soft wax bougie, passed through a canula, which being gently pressed against the anterior surface, and opening of the stricture gives, on being withdrawn, an exact representation of the diseased part. This bougie Ducamp called his "sonde exploratrice."

The dilator is now to be passed into the stricture, its presence within it may be known by the distance of the stricture from the orifice of the meatus, (previously measured by the soft bougie,) and the peculiar sensation experienced by the passage of the knobbed extremity of the central wire through the compacted and uneven portion of the urethra. Dr. Arnott sometimes introduces the dilator through a canula. As soon as the bag is sufficiently within the stricture, as much air is to be injected into it as the patient can easily bear; and during the time it remains in the urethra, the future admission or escape of the air is regulated by the sensations of the patient; that is, if the feeling of distension abate, more air may be injected, but if it should increase into pain, a little of the air may be allowed to escape.

Dr. Arnott enters largely into the evils and inconveniences arising from the use of ordinary bougies, whether by forced or gradual dilatation; and contrasts them with the advantages held out by the fluid dilator, both to patient and to practitioner. We must confess that the argument appears to us in favour of Dr. Arnott, and we are only sorry that a more extended practical application of his remedy has not been made. This we apprehend, must arise from a fancied difficulty either in the preparation or employ of the instrument. Ducamp was eminently successful in the application of his dilator, and we cannot understand why the same application has not been made of the original invention in this country. There is one evil to which we are afraid the fluid dilator is liable, although this may be avoided by limiting the size of the dilator when fully distended, and being extremely careful in the application of the distending force. The accident to which we refer is rupture of the urethra; one case of it is even related by Dr. Arnott, in which the application of the principle was made by the patient himself, "who in 1834 employed the dilator invented by Dr. Arnott; from which considerable relief was obtained. In 1831 finding the disease had returned, and that the stricture had nearly closed up again, he attempted to use the dilator himself, and in doing so had the misfortune to burst the urethra, and thus break a passage into the rectum." We suppose in this case the stricture was in the membranous portion of the urethra, and we must say that in instances where, in this situation, stricture exists, and the elasticity of the walls of the urethra are destroyed, rupture of the urethra from the employment of the fluid dilator is not unlikely, without the greatest caution, to occur.

The next mode of treating strictures is by the application of armed bougies to the diseased portion of the urethra. Mr. Wade revives the practice of Mr. Whateley, namely, the use of bougies armed with potassa fusa. This practice, very successful in the hands of the latter surgeon, had fallen into disuse, but has been again employed, and apparently with success, by Mr. Wade. The cases in which the potassa fusa may be employed with hope of success are, according to Mr. Wade, hard fibro-cartilaginous strictures, impervious to instruments without the employment of injurious pressure; hard strictures of long standing, which, although admitting the passage of a small bougie, bleed freely on its introduction; irritable strictures; spasmodic strictures, not arising from acute inflammation of the urethra; and strictures which have a marked disposition to contraction. In order to observe the effect of the potassa fusa on strictures of the urethra, Mr. Wade applied it, in the same manner as in stricture, to hard spots upon the tongue, caused by slight thickening of its mucous and submucous tissues. "The part in immediate contact with the potash quickly changes to a deep brown, surrounded by a small circle, and a little bloody serum oozes out. For two or three days afterwards the surface has a whitish appearance, and, at the end of a week or less, usually looks quite healthy; no ulceration ensues."

Mr. Wade concludes that the caustic potash acts beneficially upon strictures: first, by its dissolvent powers; secondly, by promoting absorption, and stimulating the congested vessels to contraction; thirdly, by relieving irritability and inflammation. Mr. Wade believes the potassa fusa to possess great superiority over the argenti nitras in opening a stricture. It does not occasion a slough, and hence the painful sensation produced by the urine passing over an ulcerated surface is avoided.

The following directions are given by Mr. Wade for the employment of this remedy:

"Before using the potash, a bougie should be passed down to the stricture, that its distance from the orifice of the urethra may be correctly ascertained. A small piece of potassa fusa should be inserted into a hole made in the point of a soft bougie; the eighth part of a grain is the smallest and a grain the largest quantity I am in the habit of using; but it will be rarely necessary to exceed the sixth of a grain. It will be well to make two notches on the bougie containing the potash; one marking the exact distance of the stricture; the other an inch beyond; as, very probably, on introducing the armed bougie, the first mark may be concealed within the urethra, from the penis being more stretched than when the measurement was taken. The bougie must be well moulded round the potassa fusa, so as to prevent the alkali from projecting; and it should be so placed, that it may be more applied to the upper than the lower part of the stricture for obvious reasons. Armed bougies should be well rounded at their points, to guard the urethra from the action of the potash before it reaches the stricture. In very bad cases it may be advisable occasionally to use the potassa fusa in the recumbent position, as it will then not only be best applied to the surface of the stricture, but be most likely to penetrate its texture, which, in old and hard obstructions, is very desirable. . . . To impervious strictures, from 3 to 4 are the sizes of the bougies I generally employ when using the potassa fusa; and to such as are pervious, they should be used of a size or two larger than the obstruction which the point of the instrument should penetrate. The armed bougie must be rapidly passed down to the stricture, and held against it with gentle but steadily continued pressure, for one, two, or three minutes, according to the nature of the obstruction, which if very irritable and readily bleeding, the shortest time, or even less, should be at first selected as the most prudent course." (pp. 44-5.)

In ordinary cases this remedy may be applied every second or third day. In irritable strictures it may be well to allow four days to elapse between the applications. All the irritation produced by one application must be allowed to subside before a second is resolved on. "The potassa fusa should be kept in a phial with a ground-glass stopper. It should not be broken till required for immediate use."

According to Dr. Arntzenius, in cases of strictures absolutely impervious, the practitioner has always four methods from which to choose, namely, the forcible catheterism of Dessault and Boyer, the forcible injections of Amussat, the puncture of the bladder, and the cutting into the urethra behind the stricture. Against the first, objections cannot, he holds, be too strongly stated; it is always a dangerous, often an unsuccessful, and sometimes a fatal proceeding, and the chances must always be that more harm will be done by it than good. The violent injection of warm water can be useful in very few cases, and even in the very rare instances for which (imagining them to be common occurrences,) the plan was originally invented, namely, those in which the strictured part is completely closed by a lump of mucus, the injection of water is not preferable to the introduction of a bougie, but commonly produces more pain. The puncture of the bladder above the pubis is at once the easiest and the safest operation; its danger has been much exaggerated, and it is most probable that the deaths that have followed its adoption have resulted not from its effects, but from its not having been performed sufficiently early. The puncture through the rectum is preferable to it only in the few cases in which the bladder lies very deep and far back; and the puncture through the perineum should be adopted only when effusion of urine has already taken place. The last mode of operation, by cutting into the urethra behind the stricture, is peculiarly adapted to cases in which the retention depends on obstruction by foreign bodies in the canal, or in which the urethra is manifestly very much dilated behind the stricture; but in others, the uncertainty of finding that portion of the urethra which is to be cut into, and of finding it sufficiently healthy for the introduction of an instrument, form important objections to this method. (Arntzenius, pp. 378-83.)

The propriety of these conclusions is now proved, we believe, by the experience of the best surgeons. They are followed by equally judicious observations on the treatment of some of the consequences or accompaniments of strictures, such as gleet, effusion of urine, urinary abscess and fistula, false passages, &c. Some cases are related also to prove the advantage of endeavouring to restore the urethral canal in cases of complete or nearly complete closure, by cutting into the perineum and then either cutting into or removing the strictured portion, and passing an instrument so as to connect the healthy parts of the urethra before and behind it; in other words by forming a new canal by the side of the stricture.

In a former communication in the same publication, Mr. Cooper strongly urged the superiority of the gentle treatment of stricture over the practice too frequently adopted of attempting at once to force an instrument through the obstruction. In the present, he dwells more at length on the means to be employed, and the precautions to be taken when it is determined to attempt to alleviate the symptoms of the patient by the gentle application of instruments, and lays before the profession the results of his practice on this subject, both public and private. We give additional publicity to his views of treatment, not because they are novel or original, but because his practice, which we may equally claim as ours, is judicious; and because it gains additional weight by his authority:

"It must," says Mr. Cooper, "be continually borne in mind, that all danger is not obviated by the avoidance of force; for the gentle use of the instrument may produce equally bad effects if it be injudiciously handled. On first examining the stricture, either a silver catheter or a bougie may be used for the purpose of ascertaining its seat and condition. No. 6 is the best-sized instrument for this object; it is nearly of the size of the urethra, and is much less likely than a smaller one to get entangled in the lacunæ of the canal. When the instrument is first passed down to the stricture, gentle pressure should be exercised, and, if its passage be resisted, should be continued for a few minutes. If the obstruction be owing to muscular spasm, continued pressure of three or four minutes will be sufficient to overcome the contraction; if, after that time, however, the obstacle does not yield, its persistence evidently depends upon the density of an adventitious deposition. When, in such a case, no great pain is experienced by the degree of pressure hitherto employed, and no bleeding occurs, force may be used to the full extent considered safe by the surgeons; but if this is still ineffectual, no further mechanical means should be had recourse to, (unless the bladder be much distended and the symptoms of retention urgent,) but leeches to the perinèum and a purgative draught should be prescribed. It generally happens, in cases of no very long standing, that benefit is at once derived from this treatment; and I have frequently seen the patient recover after it had been adopted for three or four alternate days; but in cases of older date, great difficulty of micturition is often found to remain, in spite of it. To combat this symptom, the application of the bougie should be continued; as it is now ascertained that the difficulty of directing the instrument into the bladder depends upon the density of the stricture, and not upon muscular contraction. A new indication now therefore presents itself, namely, the removal of the adventitious matter; this is most readily effected by maintaining an equable degree of force on the surface of the stricture, which induces inflammation and softening down of the newly-formed substance. In the course of a few days suppuration will be found to have been set up; after which five or six repetitions of the application of the instrument will be sufficient to effect its passage through the obstruction; and thus will be performed a cure of stricture in what I may term its second stage.

"It is in this second stage that the precaution which I have alluded to above is necessary; for it is requisite to be as cautious with respect to the precise direction given to the instrument when thus gently applied, as when force is used to push the catheter or sound into the bladder; inasmuch as, without proper attention to the direction of the instrument, a false passage is as likely to be produced in the one case as in the other. This I shall shortly be able to show by relating cases in point. If gentle pressure be repeatedly made against the sides of the urethra, instead of upon the stricture itself, the mucous membrane naturally yields to the continued pressure of the instrument; and a new passage is gradually produced, which by violence is frequently effected at once. When an opening has thus been formed in the urethra, it is not a necessary consequence that any train of symptoms should at once point out the error which has been committed. On the contrary, from the comparative facility with which the instrument passes onwards towards the bladder, the surgeon fancies, at first, that he has overcome the true source of obstruction, and indeed frequently succeeds in reaching the bladder with his instrument, having pushed it again into the urethra or through the prostate gland: a flow of urine then ensues, which gives him reason to suppose that the cure is complete. On the other hand, however, the urine is sometimes extravasated, as soon as the false passage has been made; an abscess is formed in the perineum, and all the symptoms follow inseparable from such a state of things, requiring, at once, the laying open of the perineum and the evacuation of the extravasated urine; and leading to the abandonment of the gentle mode of treatment for the cutting through of the permanent stricture." (Guy's Reports.)

A case is detailed by Mr. Cooper in illustration of the possibility of the occurrence of false passages even after the most gentle usage of an instrument. A gentleman, æt. 63, had been strictured for thirty years; when necessary he used bougies himself; but sometimes was so well, as not to have any occasion for them. Six or eight weeks before Mr. Cooper saw him, he discovered, after one of his aggravations, great pain and tumefaction in the perineum, which ended in extravasation of urine, and extensive sloughing of the perineum and scrotum, although all the time he made water as usual by the urethra. After deep incisions, the sloughs were cast off and the wound granulated, and at the time of making the report, he was in a fair way of getting well.

When a lengthened false passage has been made, and when no extravasation of urine has followed, it becomes difficult to ascertain that the false passage exists. False passages of the lower region are generally the result of the forcible use of instruments in or near the bulb; while those of the upper part are a consequence of the continued pressure made upon the obstruction, with the penis drawn forwards to fix the point of opposition. Sometimes the point of the instrument, after passing along the outside of the stricture, reenters the canal, and is thereby conducted into the bladder. Such an occurrence may be suspected when difficulty of passing urine returns when not relieved by the catheter, as the new canal is incapable of remaining many days pervious, unless a mucous lining membrane is formed within it; to produce which a considerable length of time is necessary. A case of this kind occurred to Mr. Cooper, in which, after having passed the instrument for weeks, in the belief that he had reestablished the natural canal, it at length, on a sudden, took a new course into the bladder, and this proved to be through the natural urethra. Mr. Cooper supposes that the temporary false passage, by diverting the urine from the back of the stricture, relieved that part from irritation, and permitted it thereby to yield and become easily permeable to the instrument. The patient was not the worse for the accident, nor was there any difficulty after in getting the instrument into the bladder through the natural canal.

Mr. Cooper recommends the caustic bougie; first, in cases which resist the application of the common bougie or catheter; and secondly, in irritable strictures. With regard to the former class, it is questionable whether everyday experience of such treatment will justify the following broad statement, namely, "that a successful result generally follows the application of this instrument." Caustic is, no doubt, in experienced hands sometimes a valuable remedy; but we cannot go along with Mr. Cooper in urging it too strongly as one for common use. If, as Mr. Cooper states, false passages are so frequently caused by unarmed instruments "as be present in most of those cases where difficulty of passage continues whilst the patient is under treatment, and that post-mortem examinations show them to be extremely common," surely armed caustic bougies, which are calculated both to produce and aggravate false passages in a tenfold greater degree in the hands of the inexperienced, should be kept far in the background. We are not yet sufficiently forgetful of the caustic mania that followed the publication of Sir E. Home's works on this subject, to lend our sanction to any fresh eulogium on the remedy calculated at all to revive indiscriminately its use. Respecting the second class to which Mr. Cooper recommends caustic bougies, viz. irritable strictures, such are admitted on all hands to be fit subjects for its use. Sir Everard Home was among the first to suggest and recommend this practice, and perhaps the sum of the remedial improvements affected on this head by this gentleman may be centered in this one point.

TRAUMATIC STRICTURES. Under this head we shall confine ourselves exclusively to the exposition of the views of Professor Franc, as detailed in the small work before us. The principal causes of this form of strictures are: 1, contusions with or without solution of continuity of the urethra in the perineal or scrotal regions; 2, lesions with or without solutions of continuity of that part of the canal which forms part of the penis; 3, internal lacerations, resulting from the careless introduction of catheters, or effected by calculi or by fragments of stone passing from the bladder.

The majority of traumatic strictures of the urethra are situated anterior to the subpubic ligament, but they are sometimes met with behind this. The part of the canal of the urethra most frequently affected is that at the anterior part of the perineum and the interval between the two sides of the scrotum. The importance of strictures resulting from injuries of the urethra depends much upon the degree of the injury which has given rise to them. These injuries may be, 1, contusion; 2, laceration; 3, loss of substance. In connexion with them also are injuries of the neighbouring parts. *Contusion* may be followed by general swelling and temporary stricture, ceasing when the swelling disappears. Induration and consequent stricture may follow the inflammation which is produced by contusion simply. *Laceration* may occur without loss of substance. It is always accompanied by swelling, and is generally followed by fistulous openings. It is not unfrequently succeeded by indurations and consequent stricture. When the laceration is from within, it is not necessarily accompanied with tumefactions. *Loss of substance* may occur in any situation and in all degrees, the character of the stricture being much influenced thereby.

*Diagnosis.* If after injury, retention of urine comes on without hemorrhage, *contusion* alone exists. If, in this case, retention is incomplete, and if during the excretion of the urine the patient feels no cutting pain where the urethra was injured, this further confirms the fact that there is neither laceration nor loss of substance. *Laceration* is characterized by hemorrhage from the urethra and pain on contact of urine. *Loss of substance* is marked by these last two symptoms, and by the formation and separation of eschars of various sizes. The cicatrices which result from the loss of substance may obliterate the canal in various degrees, and like other cicatrices they are disposed to contract. The age of the cicatrix is an important circumstance to consider in the question of the curability of stricture depending on it; the greater the age the greater the difficulty of cure.

*Treatment.* Dilatation, either continuous or intermittent, is the first means. It rarely happens that the employment of bougies is not necessary, whatever may be the degree of injury received by the canal; for in almost every instance there is at least a chronic state of engorgement of this part; and the rule of allowing the cicatrix, in cases of stricture with loss of substance, to form around a large sound is one of great importance. If this rule be neglected, the canal may be almost entirely obliterated, and if the loss of substance has affected the whole circumference of the canal, the obliteration may be complete. But if from the beginning of the treatment, when the reparatory process has but commenced, the sound be made use of, the cicatrix acquires a large superficial extent. This cicatrix in the course of time becomes lined by a new mucous membrane, in the place of that which was destroyed; that is to say, by a very thin membrane of a whitish colour secreting a mucous fluid, but destitute of follicles, glands, or villosities. The mode of conducting the dilatation must be gradual; the sound having been allowed to remain for twenty-four hours in the canal, it should be replaced either by another of a larger or of the same size. It is useless to employ the same instrument during a longer period than twenty-four hours; and the change is beneficial both by preventing its alteration by the urine and by altering the situation of its point in the bladder.

Cauterization may be employed wherever the object is to destroy a cicatrix which stretches across the canal, or a thick, old, hard cicatrix. But it is not alone sufficient to destroy the obstacle by means of caustic; it is necessary afterwards to employ dilatation, to oppose the disposition of the new cicatrix to contract, and to continue the employment of bougies until it is fair to conclude that a new mucous membrane is formed.

It is an important circumstance stated by the author (and if true of great practical application), that the cicatrices which result from wounds treated by repeated cauterization with nitrate of silver are much less retractile than others. Cauterization may also be employed where there exists a fixed pain in one part of the canal, which dilatation has not been able to modify. Cauterization will be more likely to succeed, if from the discharge of any purulent matter or other symptom the pain may be referrible to a chronic inflammation of the mucous membrane. Different circumstances will require a difference in the mode of cauterizing; thus it will be necessary to continue the cauterization longer when the object is to destroy a cicatrix which stretches across the urethra, or which is thick, hard, and old, than when it is used to repress fleshy vegetations, to resolve by means of an acute inflammation an induration of the submucous cellular tissue, or to put an end to a fixed pain which depends on chronic inflammation. In every case *lateral* cauterization must be avoided except when the canal is sufficiently large to prevent the occurrence of retention afterwards. When the contraction is too great, dilatation by bougies must always precede the use of caustic. The author appears to have had little experience in the use of scarifications and incisions, though he admits them to be occasionally necessary, nor has he anything important to say on the subject of the application of plastic surgery to deficiencies in the urethra.

According to our promise at the beginning of this article, we shall now conclude with a few brief remarks on the character of the individual works which have furnished the chief materials for its construction.

I. The Treatise of Dr. Arntzenius obtained the gold medal offered by the Society of Arts and Sciences at Utrecht for the best essay "On the diagnosis, nature, causes, consequences, and treatment of the various organic diseases of the urethra, including a critical examination of the several methods proposed for their cure, based, as far as possible, on original observations;" and there is every evidence that the prize was well awarded; for the work before us is by far more complete than any we have ever met with on the subject, and will amply supply the defect of knowledge which, the author says, has long existed in Holland respecting the diseases of the urinary passages. The whole subject is treated in the fullest and most orderly manner; the works of almost every author upon it in this country, as well as in France, Germany, and Holland, have been studied and analysed, and their most important contents are combined with the author's own observations with such rare judgment, that all appearance of the work being, as it really is in great measure, a laborious compilation, is lost. The plates are beautifully lithographed, and the paper and printing (if we may be allowed to speak of the least important things,) are such as English publishers might imitate, and are very far superior to those of any French or German work of the same character.

II. In our last Number we noticed at some length Dr. James Arnott's just claims to the discovery detailed in the work before us which was so shamefully pirated by M. Ducamp. It is, the author states, the principal object of the republication of his book, to claim priority with respect to this discovery; but, as will be seen from the notice we have taken of it, its merits are far from being confined to this. In addition to the elucidation of his own peculiar process, it contains an account of the modes practised by other surgeons in the treatment of impassable and other forms of stricture. The application of dilatation by fluid pressure to other diseases besides stricture is also considered. The author recommends its application in stricture of the rectum, enlargement of the prostate, in urinary calculi, more particularly in the female, and in obstetric surgery where it may be used to dilate the soft parts more safely than the hand. Dr. Arnott's work is a valuable surgical monograph; we are only at a loss to conceive why the plan so strongly recommended is not more universally employed.

III. Mr. Wade's book gives a short description of stricture in its various forms and stages, with their appropriate treatment. Its principal object is to hold up to our confidence the powers of the potassa fusa, whose merits appear to have been forgotten since the days of Mr. Whateley.

Although a very creditable monograph, Mr. Wade's book contains little that is new; and we think the powers of the potassa fusa might have been as well set forth in a short paper in some journal as in a volume of a hundred and forty-nine pages.

IV. M. Franc's little volume contains some valuable information on a very important subject. It is divided into two parts, the first giving a detail of cases illustrative of the observations contained in the second: it has the merit, so rare in French books, of being both small in extent and concise in style.

V. Of the general character of Mr. Cooper's papers we have already spoken: we will only here add that they are valuable contributions to surgery, and deserve the especial notice of the young surgeon.

---

